# *Ceratostylis wanrosdyi* (Orchidaceae), a new species from Peninsular Malaysia

**DOI:** 10.3897/phytokeys.279.206002

**Published:** 2026-09-24

**Authors:** Qistina Anis Mad Jabar, Edward Entalai Besi, Muhamad Ikhwanuddin Mat Esa, Sin Hoong Tan, Debbie Sandin, Christina Seok Yien Yong, Roslan Rani, Zainuddin Jamaluddin, Grippin Akeng, Nasrul Akmal Amaludin, Rusea Go

**Affiliations:** 1 Department of Biology, Faculty of Science, Universiti Putra Malaysia (UPM), 43400 Serdang, Selangor, Malaysia Department of Biology, Faculty of Science, Universiti Putra Malaysia (UPM) Serdang Malaysia; 2 Faculty of Earth Science, Universiti Malaysia Kelantan, Kampus Jeli, 17600 Jeli, Kelantan, Malaysia Faculty of Earth Science, Universiti Malaysia Kelantan, Kampus Jeli Kelantan Malaysia; 3 Jabatan Perhutanan Semenanjung Malaysia, Jalan Sultan Salahuddin, 50660 Kuala Lumpur, Malaysia Jabatan Perhutanan Semenanjung Malaysia Kuala Lumpur Malaysia; 4 Jabatan Perhutanan Negeri Pahang, Tingkat 5, Kompleks Tun Razak, Bandar Indera Mahkota, 25990 Kuantan, Pahang, Malaysia Jabatan Perhutanan Negeri Pahang Pahang Malaysia; 5 Pejabat Hutan Daerah Cameron Highlands, Tanah Rata, 39000 Cameron Highlands, Pahang, Malaysia Pejabat Hutan Daerah Cameron Highlands Pahang Malaysia

**Keywords:** Cameron Highlands, epiphytic orchids, monocots, mossy forest, new taxon, plant diversity, plant endemism, plant taxonomy

## Abstract

*Ceratostylis wanrosdyi* is described and illustrated as a new species from the mossy montane forest of Cameron Highlands, Malaysia. This epiphytic orchid is characterised by its peduncles, which are nearly as long as the leaves, and an entire, oblong-lanceolate labellum. Although the new species resembles *C. radiata* and *C. pulchella* in having white flowers, it can be distinguished by its peduncle length and labellum morphology. A detailed taxonomic description, figures, comparisons with closely related species, and a revised identification key to the species of *Ceratostylis* in Peninsular Malaysia are provided.

## Introduction

*Ceratostylis* Blume is a genus of orchids distributed from the eastern Himalayas through tropical Asia and Malesia to the western Pacific region (Teoh 2021). It currently comprises 153 species, including 12 species recorded from Peninsular Malaysia (POWO 2026), with taxa formerly placed under *Sarcostoma* Blume now included within the genus (Ng et al. 2018). *Ceratostylis* species are predominantly epiphytic orchids that inhabit lowland to montane forests and are characterised by tufted or elongated stems, fleshy leaves, terminal inflorescences, and flowers bearing eight pollinia (Seidenfaden and Wood 1992; de Leon et al. 2017). The genus name refers to the bilobed “horned” column (Greek *keras* = horn; *stylis* = style).

Recent botanical exploration in the mossy montane forests of Cameron Highlands, Pahang, resulted in the discovery of a *Ceratostylis* species with elongate peduncles nearly as long as the leaves and completely white flowers with an entire, oblong-lanceolate labellum. Detailed morphological examination and comparison with relevant literature and herbarium specimens revealed that the collected material represents a previously undescribed species. Morphologically, the new species resembles *C. eria* Govaerts, *C. radiata* J.J.Sm., *C. pulchella* Holttum, and *C. longisegmenta* Ames & C.Schweinf., all of which occur in Malaysia, but it differs markedly from them in leaf morphology, peduncle length, and labellum morphology. It also shows affinities with *C. longipedunculata* J.J.Sm., *C. anjasmorensis* J.J.Wood & J.B.Comber, *C. philippinensis* Rolfe ex Ames, and *C. pristina* M.Leon, Naive & Cootes from neighbouring regions, but can be readily distinguished by a combination of vegetative and floral characters. This taxon is therefore described and illustrated here as a new species, accompanied by comparisons with related species and a revised identification key to the *Ceratostylis* species in Peninsular Malaysia.

## Materials and methods

Morphological descriptions and measurements of the new species were based on living and preserved material. Some collected plants were cultivated *ex situ* to obtain additional floral material, while others were prepared as voucher specimens following Bridson and Forman (2000) and deposited in the Herbarium of the Department of Biology, Faculty of Science, Universiti Putra Malaysia (UPM). Flowers were preserved in Copenhagen solution (70% ethanol, 29% water, and 1% glycerol). Minute floral structures were examined using a digital microscope camera (AM4113ZT Dino-Lite Digital Microscope, Taiwan). Diagnostic morphological characters were assessed through comparison with relevant taxonomic literature (Seidenfaden and Wood 1992), herbarium specimens from UPM, KEP, and K, and digitised specimens accessed via AMES, CAL, NHN, and SING, including online records from GBIF (https://www.gbif.org). Herbarium acronyms follow Thiers (2026). Accepted nomenclature was verified using Plants of the World Online (POWO) (https://www.plantsoftheworldonline.org/). A preliminary conservation assessment was conducted following the IUCN Red List Categories and Criteria (IUCN 2024). The extent of occurrence (EOO) and area of occupancy (AOO) were estimated using GeoCAT (Bachman et al. 2011) with the IUCN-recommended default grid size of 2 × 2 km.

## Taxonomic treatment

### Ceratostylis wanrosdyi

Taxon classificationPlantaeAsparagalesOrchidaceae

Rusea, Besi & Qistina Anis sp. nov.

6F42C118-A910-5F47-B4E6-DC739858C7A6

urn:lsid:ipni.org:names:77397406-1

Figs 1, 2

#### Type.

Malaysia • Pahang, Cameron Highlands (precise coordinates withheld for conservation purposes), ca. 2,000 m elevation, 30 October 2025, *Rusea, Nasrul & Besi EDW160* (holotype: UPM! dry and wet-preserved specimens).

#### Diagnosis.

*Ceratostylis wanrosdyi* is most similar to *C. radiata*, *C. pulchella*, and *C. longisegmenta* in having white flowers with narrow petals but differs by its shorter, narrowly elliptic leaves, 2.4–3.5 × 0.3 cm (vs. narrowly lanceolate, 16.0 × 0.9 cm in *C. radiata*; linear, 12.0–20.0 × 0.5–0.7 cm in *C. pulchella*; narrowly oblong, ca. 10.5 × 1.0 cm in *C. longisegmenta*), peduncles ca. 2.3 cm long, nearly as long as the leaves at anthesis (vs. ca. 1 cm, 4 cm, and 3 cm long, respectively, and much shorter than the leaves), and an entire, broadly lanceolate labellum (vs. trilobed in *C. radiata*, *C. pulchella*, and *C. longisegmenta*).

**Figure 1.**
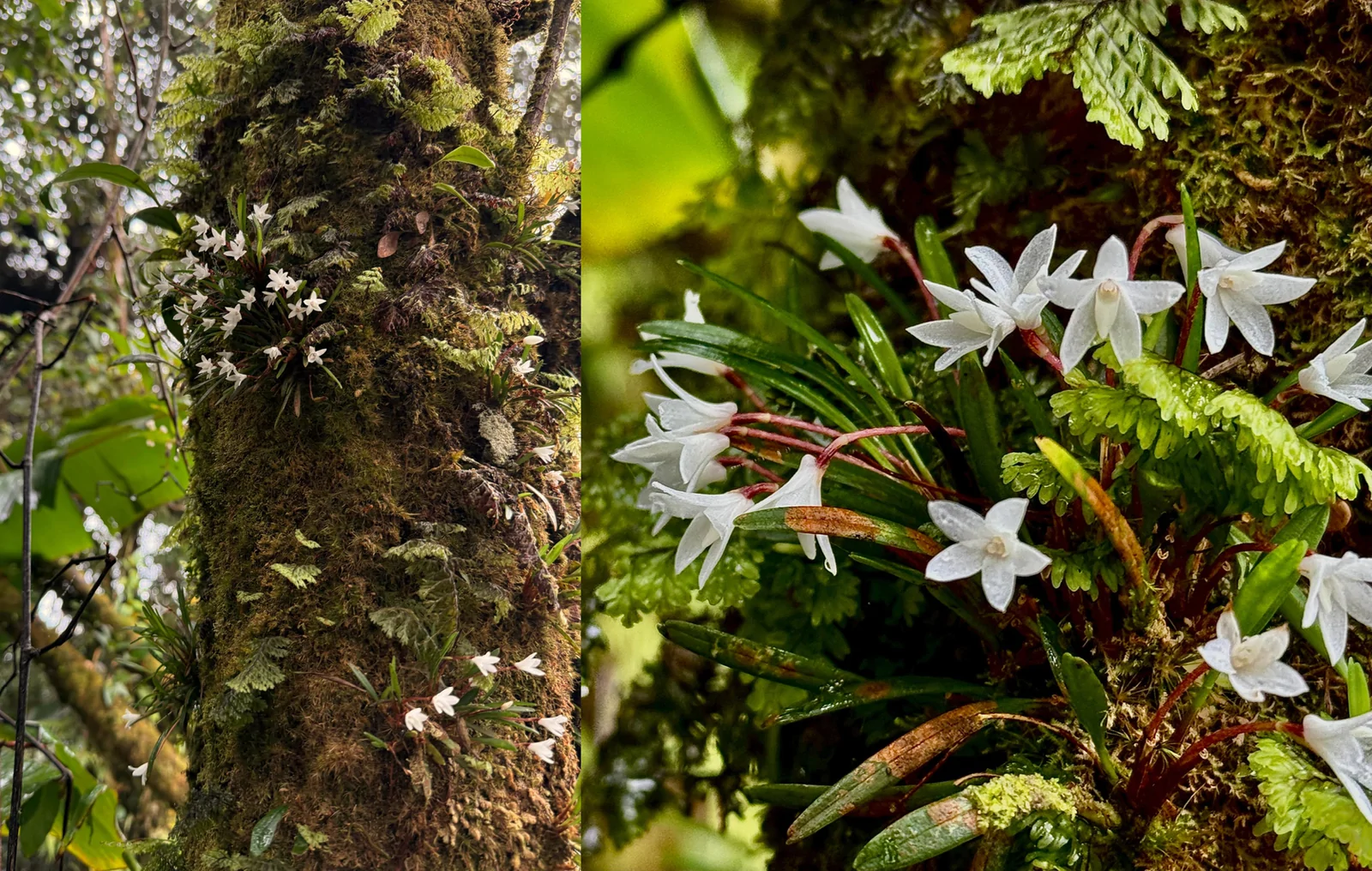
*Ceratostylis wanrosdyi* sp. nov. in its natural habitat, growing as an epiphyte in the mossy montane forest of Cameron Highlands, Pahang (type locality). Images by Rusea Go.

#### Description.

***Plant*** epiphytic, ca. 6 cm tall. ***Rhizome*** ca. 3 cm long, 0.6 mm wide, pubescent. ***Stems*** clustering, ca. 1.5–2.0 cm, covered with imbricate brown sheaths, 1- or 2-leaved; sheath narrowly lanceolate, ca. 9–12 mm long, acute to subacute, minutely punctate, veins parallel. ***Leaves*** narrowly elliptic, 2.4–3.5 cm long, 2.7–3.0 mm wide, gradually narrowing toward the base, unevenly bilobed at the apex, apex rounded to obtuse; ***petiole*** 1.0–1.3 cm long. ***Inflorescence*** terminal, single-flowered, 2.5–2.8 cm long; ***peduncle*** filiform, 2.3 cm long, 0.6 mm in diameter, reddish brown, pubescent. ***Floral bracts*** ovate, acute, 5 × 2 mm, glabrous. ***Flowers*** solitary, ca. 9.2 × 12.2 mm; **perianth** pure white, partially translucent. ***Pedicel and ovary*** 4 mm long, 1 mm diam., reddish brown, pubescent. ***Dorsal sepal*** oblong-lanceolate, 9.5 × 2.4 mm, subacute, 3-nerved. ***Lateral sepal*** elliptic-lanceolate, 10.5 × 2.9 mm, subacute, 3-nerved or 5-nerved, united basally into a blunt, globose mentum. ***Petals*** oblong-lanceolate, 9.0 × 1.7 mm, subacute, 3-nerved. ***Labellum*** entire, white, broadly lanceolate, 5.6–6.2 × 2.2–2.4 mm, 3-nerved, fleshy, basal part concave, lateral margins at basal part incurved, clawed, apical part somewhat decurved, obtuse; claw enclosed within the mentum, short, broadly falcate in outline, margin incurved. ***Column*** 1.9 mm long, base 0.9 mm wide, slightly arcuate, exalate; arms concave; foot short, conspicuous at the base; ***anther cap*** ovoid, at front emarginate, 0.8 × 0.7 mm, 2-valved; ***pollinia*** 8, pyriform, ca. 0.3 × 0.2 mm.

**Figure 2.**
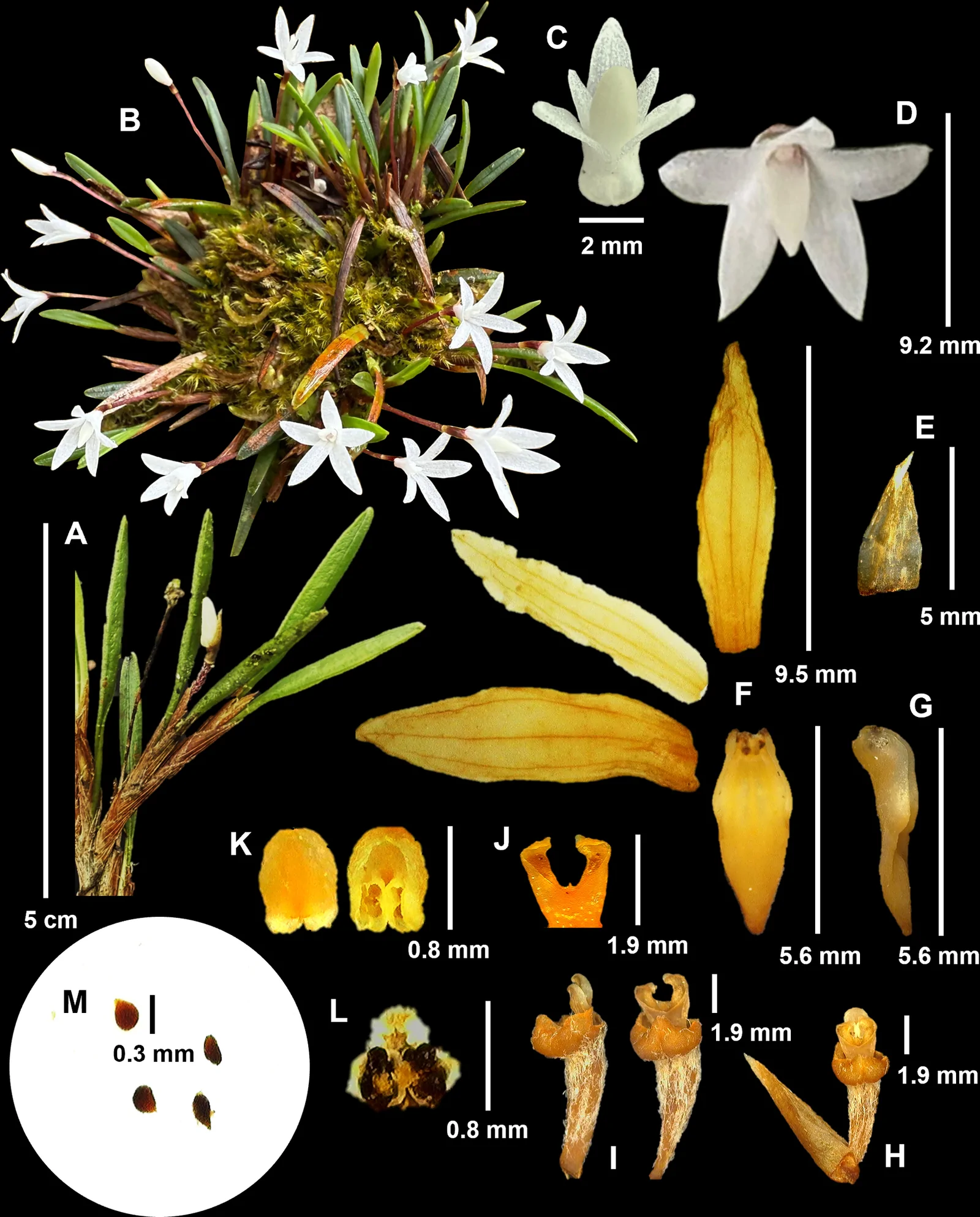
*Ceratostylis wanrosdyi***A**. Plant with flower bud; **B**. Flowering plant; **C**. Flower, view from below; **D**. Flower, front view; **E**. Floral bract; **F**. Flattened perianth segments; **G**. Labellum, side view; **H**. Column, mentum, pedicel, and floral bract; **I**. Column, mentum, and pedicel; **J**. Column apex, front view; **K**. Anther cap; **L**. Anther cap with attached pollinia, view from below; **M**. Pollinia. **E–M**. Liquid-preserved specimens. Images by Qistina Anis and Rusea Go.

#### Distribution.

The species is currently known from two localities in Cameron Highlands, Pahang, Malaysia (Fig. 3).

**Figure 3.**
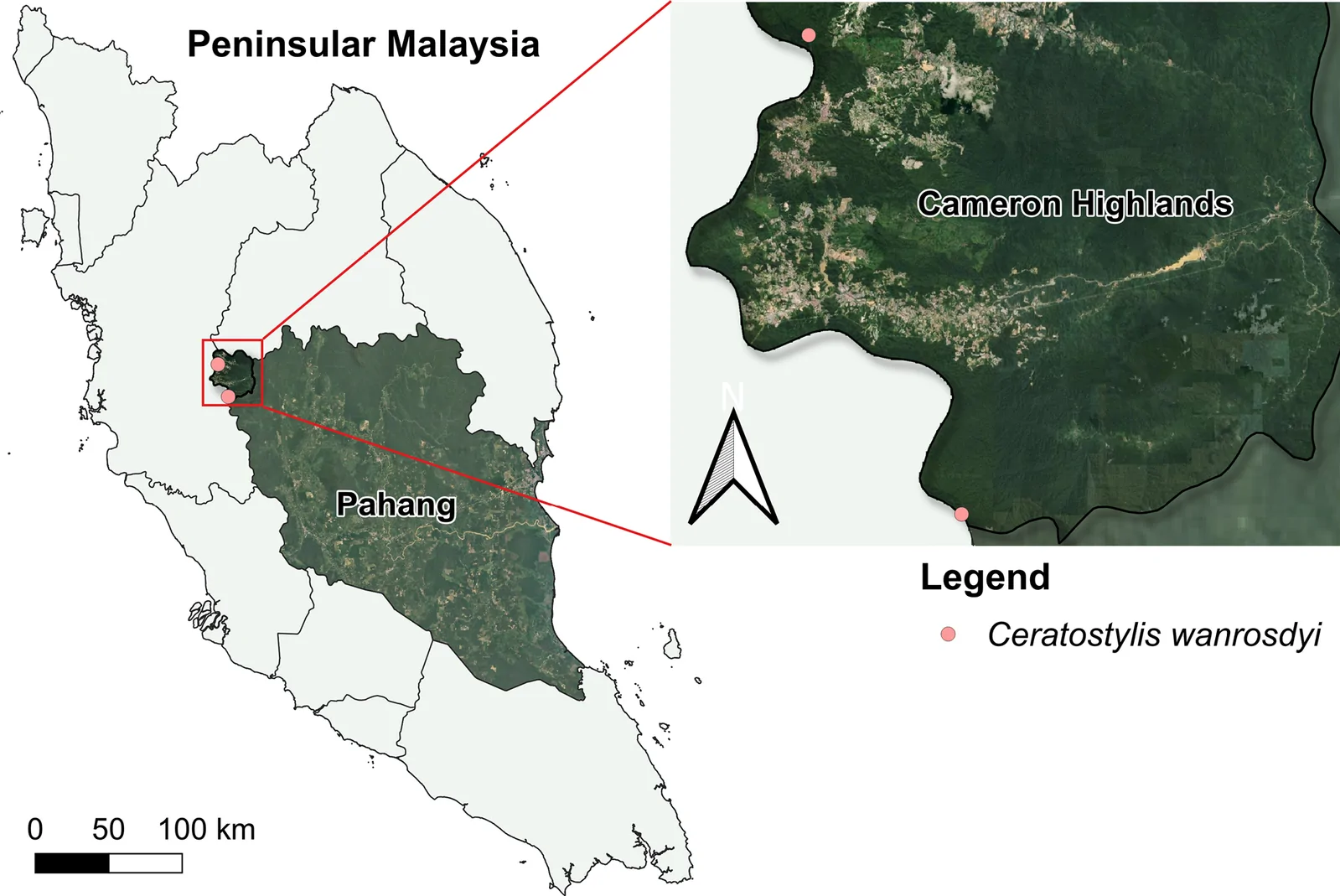
The location of *Ceratostylis wanrosdyi* in Cameron Highlands, Pahang. Owing to the potential threat of human collection, the plotted points represent approximate localities. Base map layer derived from Google Maps (Google LLC; https://www.google.com/maps).

#### Habitat.

*Ceratostylis wanrosdyi* occurs as an epiphyte in montane cloud forest at elevations of 1,800–2,000 m, on exposed mountain ridges and in partially shaded areas. The species grows on moss-covered trunks and lower branches of *Clethra hendersonii* Sleumer and several *Lithocarpus* species, including *L. elegans* (Blume) Hatus. ex Soepadmo, *L. hendersonianus* A.Camus, *L. neorobinsonii* A.Camus, and *L. suffruticosus* (Ridl.) Soepadmo. Individuals on *C. hendersonii* were observed ca. 2 m above the ground. *Ceratostylis wanrosdyi* also occurs sympatrically with *C. eria* Govaerts, another epiphytic orchid characteristic of this montane habitat.

#### Phenology.

Flowers in August and October.

#### Etymology.

The specific epithet is named in honour of the 15^th^ Menteri Besar of Pahang, YAB Dato’ Sri Diraja Haji Wan Rosdy bin Wan Ismail, in recognition of his commitment and support for biodiversity exploration, conservation, and environmental stewardship in the state. This honour also acknowledges his role in supporting and approving the proposal to establish the UNESCO Man and the Biosphere (MAB) Mossy Forest, Cameron Highlands, in 2024, which encompasses the habitat of this new species.

#### Additional specimens examined.

Malaysia • *Ceratostylis wanrosdyi*. Pahang, Cameron Highlands, 23 August 2025, *S.H. Tan & R. Go C466* (UPM!); Pahang, Cameron Highlands, 2 August 2026, *E.E. Besi, D. Sandin, & S. Mahesvaran BW001* (UPM!). • *Ceratostylis eria*. Pahang: Cameron Highlands, Summit Batu Brinchang, 1 September 1956, *H.M. Burkill 809* (K001638289!). • *Ceratostylis pulchella*. Pahang: Raub, Fraser’s Hill, January 1953, *R.E. Holttum SFN39464* (type: K000810594!). • *Ceratostylis radiata*. Kedah: Langkawi, Gunong Raya, 13 November 1921, *M. Haniff 7103* (holotype: SING0047107-image!). • *Ceratostylis longisegmenta*. Sabah, Mt. Kinabalu, Kiau, 2 November 1915, *J. Clemens 93* (holotype: AMES00061186-image!). Indonesia • *Ceratostylis longipedunculata*. Lombok: Mt. Rinjani, south side, slope above Kembang Kerang, 25 June 1909, *J. Elbert 2145* (K001638376!). • *Ceratostylis anjasmorensis*. East Java: G. Anjasmoro, 24 November 1983, *J.B. Comber 1543* (holotype: K000220023-image!). Philippines • *Ceratostylis philippinensis*. Province of Bataan Luzon: Lamao River, 21 September 1905, *H.N. Whitford 1359* (K000810579-image!); Province of Bataan Luzon, Mt. Mariveles, Lamao River, October 1903, *E.D. Merill 3247* (K000810580-image!). • *Ceratostylis pristina*. Mindanao: Bukidnon, Kalatungan Mountain Range, 21 May 2016, *M.D. De Leon MDL 007/2016* (holotype: CMUH00010858).

#### Preliminary conservation status.

*Ceratostylis wanrosdyi* is currently known from two geographically distinct subpopulations in Cameron Highlands, Pahang (Fig. 3). With only two known occurrence records, the EOO cannot be estimated meaningfully, while the AOO is estimated at 8 km^2^, corresponding to two occupied 2 × 2 km grid cells following IUCN (2024). The AOO falls within the threshold for Critically Endangered (CR) under subcriterion B2; however, the two subpopulations are treated as two locations *sensu*IUCN (2024) and therefore do not meet the CR threshold of a single location under subcriterion B2a.

Although the type locality lies within a protected forest reserve, it is situated in a popular recreational and ecotourism area that receives frequent visitor activity. The second subpopulation likewise occurs in accessible montane forest. Localised habitat disturbance associated with tourism infrastructure, trail maintenance, and recreational use is considered the main plausible threat to *Ceratostylis wanrosdyi*, as these activities could alter the humid microclimatic conditions required by epiphytic orchids. The localised nature of this threat supports the treatment of the two subpopulations as two locations *sensu*IUCN (2024), since a single threatening event affecting individuals at one location would not be expected to rapidly affect those at the other. The accessibility of both sites may also increase the risk of illegal collection, a recognised threat to wild orchid populations, particularly rare epiphytic species occurring near recreational facilities (Ballantyne and Pickering 2012; Wraith and Pickering 2017). Based on its restricted AOO, occurrence at two locations, and an inferred continuing decline in habitat quality due to ongoing anthropogenic pressures, *C. wanrosdyi* is provisionally assessed as Endangered [EN B2ab(iii)] following IUCN (2024).

#### Taxonomic notes.

*Ceratostylis wanrosdyi* shares white flowers and narrow petals with the widespread *C. radiata*, the Peninsular Malaysian endemic *C. pulchella*, and the Bornean endemic *C. longisegmenta*. The petals differ in shape among these species, being oblong-lanceolate in *C. wanrosdyi*, narrowly oblong-lanceolate in *C. radiata*, lanceolate in *C. pulchella*, and linear in *C. longisegmenta*. Differences are also evident in perianth texture: *C. radiata* has fleshy petals and a fleshy apical portion of the labellum, whereas in *C. pulchella*, both structures are membranous and as thin as the sepals. In *C. longisegmenta*, all perianth segments are thickened towards the apex, with the thickening most pronounced in the labellum (Ames and Schweinfurth 1920). In contrast, *C. wanrosdyi* has membranous sepals and petals but a fleshy labellum. A further noteworthy feature of *C. wanrosdyi* is the progressive elongation of the peduncle during floral development; it is shorter than the leaf at the budding stage (Fig. 2A) but becomes nearly as long as the leaf or slightly exceeds it at anthesis.

A further comparison with another Peninsular Malaysian endemic, *Ceratostylis eria*, reveals a superficial similarity in the elongate peduncles (ca. 3 cm long), which exceed half the length of the leaves (ca. 5.5 cm long), and in the white flowers. Nevertheless, *C. eria* is readily distinguished by its longer and wider oblanceolate leaves (ca. 5.5 × 0.5–1.2 cm), compared with the narrowly elliptic leaves of *C. wanrosdyi* (2.4–3.5 × 0.3 cm). It also differs markedly in labellum morphology and colouration. In *C. eria*, the labellum (0.4 × 0.2 cm) is much smaller than the tepals (ca. 1.4 × 0.9 cm) and is green with two dark brown blotches. In contrast, the labellum of *C. wanrosdyi* is comparable in size to the tepals, and all floral segments, including the labellum, are uniformly white.

Among species from neighbouring countries such as the Philippines, *Ceratostylis philippinensis* and *C. pristina* share white flowers and an entire labellum with the new species. However, both differ in having short peduncles, less than 1 cm long, relative to leaves measuring 1.9–3.8 cm long in *C. philippinensis* and ca. 3.0 cm long in *C. pristina*. Moreover, *C. wanrosdyi* is distinguished from *C. philippinensis* by its larger petals (oblong-lanceolate, 9.0 × 1.7 mm vs. narrowly lanceolate, 5.0 × 1.0 mm) and labellum (broadly lanceolate, 5.6–6.2 × 2.2–2.4 mm vs. lanceolate to broadly lanceolate, ca. 4.0 mm long). It further differs from *C. pristina* in petal morphology (oblong-lanceolate, three-nerved vs. ovate, five-nerved) and labellum shape (broadly lanceolate vs. ovate).

The new species also resembles *Ceratostylis longipedunculata* from the Lesser Sunda Islands (Bali and Lombok) and *C. anjasmorensis* from Java in having peduncles nearly as long as the leaves. Nevertheless, *C. wanrosdyi* differs markedly from *C. longipedunculata* in floral colouration (pure white vs. white with a purple tint) and labellum morphology (broadly lanceolate, pure white vs. spathulate, white at the base and yellow from the centre towards the apex). Compared with *C. anjasmorensis*, *C. wanrosdyi* has larger petals (oblong-lanceolate, 9.0 × 1.7 mm vs. narrowly elliptic, 0.8–1.0 × 0.5 mm) and a larger labellum (5.6–6.2 × 2.2–2.4 mm vs. 3.0 × 0.8 mm). The two species also differ in floral colouration. All perianth segments of *C. wanrosdyi*, including the labellum, are uniformly white, whereas in *C. anjasmorensis*, the sepals and petals are translucent white, tinged pink towards the apices, while the labellum is white at the base and yellow towards the apex (Wood and Comber 1986).

A comparative summary of the new species with selected congeners is presented in Table 1. The comparison includes species with predominantly white, star-shaped flowers, namely *C. eria* (Hooker 1891; Seidenfaden and Wood 1992), *C. radiata* (Smith 1905; Seidenfaden and Wood 1992; Odyuo et al. 2013), *C. pulchella* (Holttum 1953; Seidenfaden and Wood 1992), *C. philippinensis* (Ames 1905), and *C. pristina* (de Leon et al. 2017), together with *C. longipedunculata* (Smith 1925; Atmaja and Tirta 2019), which is included because of its similarly elongate peduncles. Morphological data for the selected congeners were compiled from the respective literature cited above and verified against herbarium specimens examined in this study wherever possible.

**Table 1.** Diagnostic morphological comparison of *Ceratostylis wanrosdyi* and closely related species.

**Characters**	***Ceratostylis wanrosdyi***	***C. eria***	***C. radiata***	***C. pulchella***	***C. philippinensis***	***C. pristina***	***C. longipedunculata***
Sheath venation	Parallel	Parallel	Reticulate	Reticulate	Parallel	Reticulate	Reticulate
Leaf shape	Narrowly elliptic	Narrowly oblanceolate	Narrowly lanceolate	Linear	Linear	Linear	Narrowly lanceolate
Leaf size	2.4–3.5 × 0.3 cm	5.5 × 0.5–1.2 cm	16.0 × 0.9 cm	12.0–20.0 × 0.5–0.7 cm	1.9–3.8 cm long	3.0 × 0.3 cm	6.3–9.0 × 0.8 cm
Leaf apex	Unevenly bilobed	Unevenly bilobed	Unevenly bilobed	Acute	Subacute	Unevenly bilobed	Emarginate
Peduncle length	2.3 cm	3.0 cm	1.0 cm	4.0 cm	Less than 1.0 cm	ca. 1.0 cm	1.2–2.0 cm
Flower tepal colour	White	White	White	White, translucent	White	White, partially translucent	White with purple tint
Petal shape, size and nervation	Oblong-lanceolate, 9.0 × 1.7 mm, subacute, 3-nerved	Oblanceolate, 9.0–14.0 × 5.0 mm, acute, 5-nerved	Narrowly oblong-lanceolate, 8.0–13.0 × 2.0–2.5 mm, acute, 3-nerved	Lanceolate, 11.0 × 2.0 mm, acute to subacute	Linear-lanceolate, 5.0 × 1.0 mm, subacute, 3-nerved	Ovate, 6.0 × 3.0 mm, subacute, 5-nerved	Narrowly lanceolate, ca. 5.0 × 0.7 mm, obtuse, 1-nerved
Labellum shape and size	Entire, broadly lanceolate, 5.6–6.2 × 2.2–2.4 mm	Entire, lanceolate, 4.0 × 2.0 mm	3-lobed, spathulate, 12.5–17.0 × 2.0–5.0 mm; lateral lobes triangular	3-lobed, lanceolate, narrowed toward apex, 9.0 × 3.0 mm; lateral lobes small	Entire, lanceolate to broadly lanceolate, 4.0 mm long	Entire, ovate, 2.3 mm × 2.0 mm	Entire, spathulate, widened toward apex, 6.6 mm long
Labellum colour	White	Green with two dark brown patches at base	White	White	White	White	White at base, yellow from centre towards the apex

##### Revised artificial key to the species of *Ceratostylis* in Peninsular Malaysia, adapted from Seidenfaden and Wood (1992)

**Table:** 

1	Stem rush-like, green, naked, sheathed at the base only	**2**
–	Stem not rush-like, entirely or almost entirely covered with sheaths	**4**
2	Leaf 1.5–4 cm long, less than one-third the length of the stem, almost terete	**3**
–	Leaf up to 8.5 cm long, as long as or longer than the stem, distinctly flattened	***C. gracilis***
3	Stem approximately 7–15 cm long. Mentum 1.5 mm long. Sepals and petals yellow or pale purple	***C. subulata***
–	Stem approximately 20–35 cm long. Mentum 2–3 mm long. Sepals and petals deep purple	***C. ampullacea***
4	Stem closely aggregated	**5**
–	Stem loosely spaced, ca. 1 cm apart	**6**
5	Stem approximately 10 cm long	***C. lancifolia***
–	Stem very short, approximately 1 cm long	**7**
6	Leaf to 12 by 2.5 cm	***C. robusta***
–	Leaf shorter, 1.5–6 cm long	**8**
7	Leaf approximately 5.5 by 1 cm, not over 6 cm long, linear-oblanceolate	***C. eria***
–	Leaf over 6 cm long, linear or linear-lanceolate	**9**
8	Sheaths without distinct venation, minutely punctate	***C. puncticulata***
–	Sheaths distinctly veined, parallel or reticulate	**10**
9	Petals white with deep crimson along margins of basal half. Dorsal sepal oblong	***C. javanica***
–	Petals entirely white. Dorsal sepal lanceolate	**11**
10	Peduncle elongate, up to 2.3 cm long, equal or more than three-quarters of the leaf length	***C. wanrosdyi***
–	Peduncle ca. 0.5–1 cm long, less than one-half of the leaf length	**12**
11	Labellum hairy on the inner surface; lateral lobes finely ciliate	***C. radiata***
–	Labellum glabrous	***C. pulchella***
12	Flowers white. Labellum apex acute	***C. pendula***
–	Flowers yellow with dark purple lines. Labellum apex truncate	***C. clathrata***

## Supplementary Material

XML Treatment for Ceratostylis wanrosdyi

## Additional information

### Artificial Intelligence (AI) use

The authors accept full responsibility for the content of the manuscript, including the disclosure of any use of AI.

No AI tools were used in the preparation of this manuscript.

### Author ORCIDs

Q.A. Mad Jabar https://orcid.org/0009-0004-9428-8769

E.E. Besi https://orcid.org/0000-0002-1394-1008

M.I. Mat Esa https://orcid.org/0000-0003-4728-6109

S.H. Tan https://orcid.org/0000-0002-4021-0331

C.S.Y. Yong https://orcid.org/0000-0002-1399-971X

R. Go https://orcid.org/0000-0003-3922-5082

### Data availability

All of the data that support the findings of this study are available in the main text.
